# Student experiences of participating in five collaborative blended learning courses in Africa and Asia: a survey

**DOI:** 10.3402/gha.v9.28145

**Published:** 2016-10-06

**Authors:** Salla Atkins, Weirong Yan, Elnta Meragia, Hassan Mahomed, Senia Rosales-Klintz, Donald Skinner, Merrick Zwarenstein, for the ARCADE consortium

**Affiliations:** 1 Department of Public Health Sciences (Global Health/IHCAR), Karolinska Institutet, Stockholm, Sweden; 2 Tongji Medical College, Huazhong, University of Science and Technology, Wuhan, China; 3 Division of Community Health, Department of Interdisciplinary Sciences, Faculty of Medicine and Health Sciences, Stellenbosch University, Stellenbosch, South Africa; 4 Metro District Health Services, Western Cape Government, Cape Town, South Africa; 5 Research on Health and Society, Faculty of Medicine and Health Sciences, Stellenbosch University, Tygerberg, South Africa; 6 Department of Family Medicine, Centre for Studies in Family Medicine, Schulich School of Medicine & Dentistry, Western University, London, ON, Canada

**Keywords:** blended learning, capacity building, global health, student experience

## Abstract

**Background:**

As blended learning (BL; a combination of face-to-face and e-learning methods) becomes more commonplace, it is important to assess whether students find it useful for their studies. ARCADE HSSR and ARCADE RSDH (African Regional Capacity Development for Health Systems and Services Research; Asian Regional Capacity Development for Research on Social Determinants of Health) were unique capacity-building projects, focusing on developing BL in Africa and Asia on issues related to global health.

**Objective:**

We aimed to evaluate the student experience of participating in any of five ARCADE BL courses implemented collaboratively at institutions from Africa, Asia, and Europe.

**Design:**

A post-course student survey with 118 students was conducted. The data were collected using email or through an e-learning platform. Data were analysed with SAS, using bivariate and multiple logistic regression. We focused on the associations between various demographic and experience variables and student-reported overall perceptions of the courses.

**Results:**

In total, 82 students responded to the survey. In bivariate logistic regression, the course a student took [*p*=0.0067, odds ratio (OR)=0.192; 95% confidence interval (CI): 0.058–0.633], male gender of student (*p*=0.0474, OR=0.255; 95% CI: 0.066–0.985), not experiencing technical problems (*p*<0.001, OR=17.286; 95% CI: 4.629–64.554), and reporting the discussion forum as adequate for student needs (*p*=0.0036, OR=0.165; 95% CI: 0.049–0.555) were found to be associated with a more positive perception of BL, as measured by student rating of the overall helpfulness of the e-learning component to their studies. In contrast, perceiving the assessment as adequate was associated with a worse perception of overall usefulness. In a multiple regression, the course, experiencing no technical problems, and perceiving the discussion as adequate remained significantly associated with a more positively rated perception of the usefulness of the online component of the blended courses.

**Discussion:**

The results suggest that lack of technical problems and functioning discussion forums are of importance during BL courses focusing on global health-related topics. Through paying attention to these aspects, global health education could be provided using BL approaches to student satisfaction.

## Introduction

Demand for education in global health, including global health research, is increasing worldwide (1–5). Education in global health research can contribute to improving the health status of low- to middle-income countries (LMICs) by training a cadre of health researchers who can provide timely, relevant evidence to policymakers for improving health systems (4, 6). The need and demand for global health research education may be difficult for LMIC academic institutions to meet, as institutional capacity for education and research continues to be low (6–8) despite many efforts to encourage and support capacity building (9–11). Because of the mismatch between global health research education demand (12) and the capacity to provide training courses (7), innovative approaches are needed.

One approach to address the gap in the availability of global health research education has been through the application of e-learning. While e-learning may be exclusively online (or computer based), with no face to face classroom contact, a newer approach, called blended learning (BL), uses web-based technology to facilitate learning and teaching both inside *and* outside the classroom (12–14). By replacing some in-person lectures with didactic materials to be viewed online or after downloading, BL makes it possible to focus real-time contacts on genuinely interactive learning, such as seminars or tutorials. This approach allows institutions to offer learning experiences to more students (15) than in traditional, face-to-face only, campus-based education and thus has potential to alleviate the shortage of faculty in LMICs (7, 8, 12, 15). Other benefits to students include, for example, being able to access learning materials from the comfort of their own home and setting their own learning schedule, potentially gaining flexibility (16). BL can link entire classrooms at different universities and even different countries in identical, similar, or merely overlapping courses and for real-time discussions. This approach can have benefits for student learning (17) especially in relation to global health.

The BL approach was tested recently in the ARCADE HSSR and ARCADE RSDH (African Regional Capacity Development for Health Systems and Services Research; Asian Regional Capacity Development for Research on Social Determinants of Health) projects (18). Other articles in this special issue have explored the experience of the ARCADE projects in terms of the resources and infrastructure needed (19), costs from an institutional perspective (20), and from the points of view of the lecturers who are key to implementation (21). However, a key question in establishing whether BL could contribute to building capacity in LMICs relates to the views of the students involved and their experiences of the courses implemented, particularly since student perceptions of BL courses are associated with their educational achievements (22, 23).

Existing studies indicate that students find BL courses flexible and convenient (24) and that BL courses can offer more of a sense of community to students than the individualistic and often isolated pure e-learning experience (25). Although several aspects of BL have been studied, few have investigated student experiences of (26) and attitudes toward BL, and rarely using survey methods (27). Blended learning is dependent on online platforms and online interaction for its success, and it is important to know whether students, having experienced such courses, found these aspects useful. Studies focusing on these aspects are particularly lacking from LMICs, settings with a lower level of technological infrastructure, and in the context of a global networking project such as ARCADE.

We surveyed student experiences of five collaborative BL courses in Asia and Africa that were delivered under the framework of the ARCADE projects (28). We aimed to investigate what is associated with student perceptions of the usefulness of online learning platforms for their studies when global health-related courses are implemented through BL, to inform the development of future BL courses.

## Methods

### The project: participating universities, course offerings

The ARCADE projects focused on research capacity building in health systems and services in Africa (ARCADE HSSR) and on building capacity for research into social determinants of health in Asia (ARCADE RSDH), funded under the seventh framework of the European Community (18). The projects involved 16 partners in total across Africa, Asia, and Europe. These partnerships developed courses on health research methods in a variety of topic areas with the purpose of enabling training of health researchers in LMICs [for more details about the projects see (18) in this issue].

The courses developed by the consortia were delivered using different methods according to the needs of the participants and the available infrastructure. Exploiting the existing technological possibilities, the courses were run either through pure e-learning (self-learning through digital means) or using BL methods (a combination of face-to-face and self-learning). The partners in each consortium were universities, research institutes, or medical colleges in China, Finland, India, Malawi, Norway, Oman, South Africa, Sweden, Tanzania, Uganda, the United Kingdom, and Vietnam.

This paper focuses on the evaluation of five courses developed in these projects (see Table 1 for a summary of the courses).

**Table 1:** Summary of the courses under evaluation

Course code Title – universities involved (Order of course in ARCADE courses)	Short description of the course	Mode of delivery	Assessment methods	Participating students (*n*)
Course 1 Improving Drug Use, Especially Antibiotics – KI; Ujjain Charitable Trust Hospital and Research Centre, India; Tongji Medical College (TJMC), Huazhong University of Science and Technology (HUST), China; Hanoi Medical University (5)	The course introduced the scope and main methods of drug utilisation research, methods to estimate the level of self-medication, and people's beliefs and behaviour, with a particular focus on antibiotics	One week full-time studies (1.5 European Credit Transfer System (ECTS) credits). The course offered a combination of synchronous real-time interactive lectures and recorded lectures available via Ping Pong	Individual assignment describing an issue of antibiotic use or antibiotic resistance and suggestions for action	21
Course 2 Meta-Analysis of Diagnostic Accuracy Tools (MADAS) – MU, SU, KI (1)	The course was designed to teach students how to conduct a meta-analysis of a diagnostic accuracy study – from study design to manuscript preparation	One week full-time (1.5 ECTS). Almost 50% synchronous teaching. Lectures were recorded during the session and posted on the Moodle platform The rest of the course focused on reading articles shared on Moodle and practical training in meta-analysis	Multiple-choice exam	20
Course 3 Practical Approaches to Qualitative Research – SU, MU, KI (3)	The course aimed to give a general practical basis to researchers wishing to learn qualitative research methods	Thirteen weeks, part-time (7.5 ECTS). Students viewed lectures and read articles based on Moodle for 12 weeks and used a discussion forum. The course also included 1 week of face-to-face skill training at each site focusing on practical skills	Written assignments and participation in online discussions	27
Course 4 Qualitative Evaluation in Health Care – KI, Malawi University, Tongji Medical College of HUST (4)	The course focused on qualitative evaluation methods for health systems and services research. It provided a theoretical and practical orientation to qualitative evaluation	Two weeks (part-time, 1.5 ECTS), with 1 week of synchronous real-time online lecturing and recorded lectures available via Moodle and 1 week of self-study and project work to develop an evaluation protocol	Study protocols developed and multiple-choice quiz	12
Course 5 Randomized Controlled Trials – SU, KI, MU (2)	The course covered the principles of clinical trials in investigating effectiveness, efficacy, and safety of treatments/interventions	The course was given over 10 weeks at SU, and a more intensive version (1 week full-time studies) was offered over the last week at KI and MU. Students from all three sites participated in real-time lectures and question and answer sessions with an expert	Progressively developed protocol of an RCT through three assignments, an online quiz, and an examination	38

Adapted from Ref. (21). KI, Karolinska Institutet; MU, Makerere University; SU, Stellenbosch University.

## Data collection

### Study design

This was a cross-sectional study, using a quantitative student survey with closed-ended questions.

### Study population

The study population included 118 students who participated in ARCADE courses in South Africa, Uganda, Sweden, China, India, and Vietnam in 2012–2013.

### Data collection

A questionnaire was developed based on a review of relevant literature and existing student evaluation questionnaires (see Appendix 1). The questionnaire was assessed by members of the ARCADE collaboration and face validity was established. Various forms of the questionnaire were adopted depending on the course content and host institution needs. The questionnaire was sent to all students after completion of their course using email, online discussion platforms, or by mail. They were reminded at least twice to respond. The questionnaire included questions on various aspects of the course, such as experiences of the e-learning platforms used and their experience of attending the course. Students were asked to rate various aspects of the course using a Likert-scale response of 1–6 (1=strongly disagree, 6=strongly agree^1^), in addition to yes/no questions (see Appendix 1 for the questions included in this study). Four of the courses collected additional detailed demographic information from students.

### Data analysis

Data that were common to all or most of the courses were extracted into Excel worksheets and analysed using SAS. Descriptive statistics were calculated for all variables. Thereafter, bivariate logistic regression was performed to study associations between key data items and the reported overall experience of using the online platform. The question used to indicate the general experience of BL was, ‘Overall, I feel that the online platform has helped me in my studying’. As a final stage of analysis, variables found to be significantly associated with student self-rated overall experience of the platform and other variables of potential interest were entered as independent variables into a multivariate logistic regression analysis. An alpha level of *p<*0.05 was considered statistically significant. The best-fitted regression model was used to determine the main factors influencing the overall experience variable.

### Ethical considerations

Students were informed of the evaluation when they participated in classes. The survey was filled out on a voluntary basis, and no identifying information was captured. The students were made aware that participation had no impact on their course evaluations. The survey had ethical approval from the University of Stellenbosch (SU), South Africa.

## Results

Of the total 118 students that took part in the courses, 82 students (69.5%) responded to the evaluation survey. Table 2 details the demographics of the respondents. Of these, 33 (40.2%) were male and 47 (57.9%) were female, while three did not report their sex. The mean age of students in the four courses for which data were collected was 34.8 years, with students in the RCT course somewhat younger on average. All courses had more female than male students, again with the exception of the RCT course. This RCT course also catered for master's students at SU, who made up the majority of the class. Only one student reported a disability.

**Table 2:** Student demographics

Variables	*n* (%)
Sex	
Male	33 (40.2%)
Female	47 (57.3%)
Missing	2 (2.4%)
Age (years)	34.8 (average %)
< 40	47 (57.3%)
≥ 40	14 (17.07%)
Missing*	21 (25.6%)
Number of adults in your household	2.4
< 3	30 (36.5%)
≥ 3	14 (17.0%)
Missing	38 (46.3%)
Number of children in your household	1.25 (average %)
Children <5	
Yes	29 (35.3%)
No	32 (39.0%)
Missing	21 (25.6%)
Combine work with studies	
Yes	49 (59.7%)
No	9 (10.9%)
Missing	24 (29.2%)

Table 3 details the variables in the analysis, showing means and standard deviations.

**Table 3:** Variables, mean, and standard deviation

Variable	*n*	Mean	SD	Answer scales
Overall the e-learning platform helped me with studying	78	4.72	1.32	Rating 1–6
How often do you access the online site?	78	3.68	0.97	Rating 1–6
The e-learning platform is easy to use	78	4.79	1.18	Rating 1–6
I experienced few or no technical problems accessing the platform	78	4.54	1.51	Rating 1–6
Ranking of helpfulness: e-learning platform	39	3.28	0.86	Rating 1–4
Ranking of helpfulness: information*	76	2.91	1.04	Rating 1–4
Ranking of helpfulness: content*	76	3.29	0.86	Rating 1–4
Ranking of helpfulness (1–4): communication*	75	2.6	1.08	Rating 1–4
Ranking of helpfulness (1–4): assessment*	75	2.95	0.93	Rating 1–4
The discussion forums on the e-learning platform were adequate to be able to share information and extend my understanding of the materials in the course	67	3.93	1.5	Rating 1–6
Did you experience problems with the discussion forums?	67	0.43	0.5	Yes/no
In future, would you like to see further development of and delivery through online self-study?	70	2.41	0.86	Rating 1–3 1: yes, more online 2: no, more face to face 3: happy with the current mix

Table 4 shows the associations between the independent variables and overall experience of the online learning platform from the bivariate logistic regression analysis.

**Table 4:** Association between overall experience of the online learning platform and selected independent variables

Variable	Point estimate (odds ratio)	95% confidence interval	*p*
Course 1	>999.999	<0.001– > 999.999	0.9652
Course 2	0.192	0.058–0.633	0.0067
Course 3	2.644	0.543–12.879	0.2286
Course 4	0.552	0.125–2.426	0.4311
Course 5	1.637	0.414–6.467	0.4819
Gender	0.255	0.066–0.985	0.0474
Age	2.053	0.225–18.686	0.5234
Number of adults	2.954	0.310–28.135	0.3462
Number of children	0.846	0.136–5.278	0.8581
Children <5	0.554	0.119–2.571	0.4506
Home Internet connectivity	0.200	0.025–1.587	0.0002
Combine studies with employment	2.048	0.342–12.247	0.4322
Easy to use	0.965	0.184–5.058	0.9660
No technical problems	17.286	4.629–64.554	<0.0001
E-learning platform	1.260	0.250–6.350	0.7794
Information	0.778	0.221–2.740	0.6957
Content	0.297	0.035–2.492	0.2634
Communication	0.470	0.145–1.521	0.2077
Assessment	0.121	0.015–0.978	0.0477
Adequacy of discussion forum	0.165	0.049–0.555	0.0036
Problems with discussion forum	0.356	0.112–1.137	0.0813
More online learning	0.667	0.097–4.579	0.3032
Less online learning	1.850	0.303–11.295	0.1924

The results showed that variables significantly associated with a perception that the BL platform had not been helpful to studying included the course attended being Course 2 (Meta-Analysis of Diagnostic Accuracy Tools (MADAS)) [*p* = 0.0067, odds ratio (OR)=0.192; 95% confidence interval (CI): 0.058–0.633] and perceiving the assessment as useful (*p=*0.0477, OR=0.121; 95% CI: 0.015–0.978). Gender also emerged as significant (*p=*0.0474, OR=0.255; 95% CI: 0.066–0.985), with female students ranking the helpfulness of the platform for their studies lower. Of experiences specific to the courses, not experiencing technical problems (*p<*0.001, OR=17.286; 95% CI: 4.629–64.554) was associated with a better overall experience of the platform. In addition, perceiving the course discussion forum as adequate was associated with a better rating of the helpfulness of the platform (*p=*0.0036, OR=0.165; 95% CI: 0.049–0.555).

We knew that teaching methods, topics, instructors, tutors, and delivery methods differed between courses and suspected that these differences could affect the relationships between the other independent variables and the overall experience. Thus we entered the significant factors into a multiple logistic regression analysis to account for interactions between the variables and controlling for course. Table 5 presents the results of the multivariate analysis.

**Table 5:** Multivariate logistic regression of association between key variables and reported overall experience

Effect	OR	95% Wald confidence limits	*p*
Course 2	0.019	<0.001–0.511	0.0183
Gender	0.460	0.057–3.739	0.4677
Assessment	0.067	0.002–2.273	0.1327
No technical problems	18.500	1.567–218.482	0.0205
Adequacy of discussion forum	0.018	0001–0.298	0.0051

In the multiple regression, participating in Course 2 predicted a worse opinion of the helpfulness of the online component (*p=*0.0183, OR=0.019; 95% CI: <0.001–0.511). Perceiving the course discussion forum as adequate was significantly associated with a positive overall experience of the platform (*p=*0.0051, OR=0.018; 95% CI: 0.001–0.298) suggesting that persons more satisfied with the course discussion forum reported a higher overall satisfaction with the BL platform in general. In addition, not experiencing technical problems was associated with a better rating of the course (*p* = 0.0205, OR = 18.500; 95% CI: 1.567–218.482), though the confidence interval is extremely wide.

## Discussion

In our multiple regression analysis, we identified that the main factors associated with a positive overall experience of ARCADE courses’ online platforms, and thus possibly the online component of the courses, were the course the students had attended, perceiving the discussion forum as adequate, and having experienced no technical problems. Of all the courses evaluated, course 2, the MADAS course that was the first instance of an ARCADE course, was reported by students as having a poorer overall experience of the platform than respondents taking part in the other courses. As seen in Table 1 and reported elsewhere (29), the MADAS course was the first attempt by the ARCADE projects at implementing BL. Students and staff experienced significant technical problems during its implementation and the course had less focus on using the online platform. Although it is disappointing that one of the courses could stand out as having a negative effect on satisfaction with online components of the course, it is encouraging that the other courses fared better in the analysis, suggesting that the consortia learned as the projects progressed. It should also be noted that this course made little use of the platform and that the main sharing of ideas was in real time and communication via email, making it not truly a blended course.

The perceived adequacy of the discussion forum was significantly associated with the perceived helpfulness of the BL platform. This finding is not wholly surprising, as interaction is considered key to learning (30) and both interaction with lecturers and interaction with other students are considered important (31). Some of the ARCADE courses utilised online discussions, with students posting comments and awaiting a response some time later; others preferred real-time discussions, and some used a blend of the two. We did not explore this issue in detail, but our results do highlight the importance of an opportunity to discuss learning with peers and tutors and the importance of the discussion forum component of BL. Our evaluation of the experience of lecturers suggests that they found many of the discussion formats challenging, either in terms of time or technology (21). Further work should be conducted on how to establish a high quality discussion forum even when conducting BL across several institutes.

The findings also highlight the importance of considering student capabilities and the environments from which they come, including technological surroundings, when implementing BL for global health research education. Not experiencing technical problems was significantly associated with a more positive perception of the overall usefulness of the online component of the course. Unfortunately, the data collected did not identify the country in which the student participated in the course – for example, many Karolinska Institutet students could take part from India or Uganda. Across different settings, students experienced different challenges with technology, from electricity blackouts to lack of skills necessary to learn efficiently online. Online learning can also require from students a different level of self-directedness and activity, for which IT skills are key (32). Should students’ IT skills not meet the requirements for the course, they may perceive the course as not useful for their learning. Further, ARCADE courses could be conceptualised as originating from constructivist learning (33), with a strong focus on interaction and discussion. The students were asked to rate the usefulness of the online component for their course in the survey. As the focus of many of these courses was on the communal construction of knowledge, it could be that the online component was not useful for the students’ learning, particularly when discussions were already in classrooms or live or when technical problems were present.

Our analysis and data cannot answer all the questions around student IT skills and how both technological and infrastructural contexts and cultural perceptions of education and interactions with both instructors and peers outside the online platform impact on their reported experiences. Further qualitative research, along with student research with stronger study designs such as randomized trials, could contribute toward exploring these issues. Further work should be directed at countering these challenges.

This study has a number of limitations. First, the sample size was small for the number of courses, and our analysis was not strong enough to account for the clustering of the responses. Second, we could not capture effects relating to the institution or country the students were from. Third, the level of missing data for some independent variables limits the reliability of the findings.

## Conclusions

This small-scale study explored the perceptions of students participating in multicountry courses on global health–related subjects through BL. Experiencing technical problems during the course and perceiving the discussion forum as inadequate had a significant relationship with the overall perception of the online course platform as not useful. These factors may be dependent not only on students’ level of computer skills and familiarity with the Internet, but also on the available time and interest they have in engaging in BL. Future iterations of BL should ensure that students receive adequate support in engaging with courses and that facilities for online discussions are considerably improved.

## Footnotes

1 For one variable, *adequacy of discussion*, this rating scale was flipped, with 6 indicating *strongly disagree*.

## Paper context

The ARCADE projects were research capacity building projects in Africa and Asia, where there remains a dearth of policy relevant research. One of the ways in which capacity was built was through delivering blended (online combined with face to face instruction) courses. This article describes student experiences of various aspects of the courses.

## Appendix 1. Extract from the student questionnaire

Demographic/profile information Male (M)/female (F) Your age _______ Do you have a disability? Yes (Y)/no (N) If yes, please specify _________ Number of adults residing in your home including you. Number of children residing in your home. Do you have children under 5? Yes (Y)/no (N) If yes, please specify how many _________ Do you have Internet connectivity at home? Yes (Y)/no (N) Do you combine your studies with employment? Yes (Y)/no (N) Please specify what kind of job you have ____________ Academic work/teaching at university (A) Research (not related to your course research) (R) Clinical work (C) Project work (P) Work at laboratory (L) Other (O), please specify: ___________________ Level of effort in your work,% (e.g. for part-time employment 30% or 50% or 100% for full-time employment) ____________ In what capacity did you attend this course? Master's student (M), doctoral student (D), postdoc (PD), other (O), please specify: __________________ Please specify which university you attend: Experience with e-learning: 1. Overall I feel that the e-learning platform (xxxxx) has helped me to learn during the course. (1 = strongly disagree; 6 = strongly agree) 2. How often have you gone online to access the course materials during this course? Several times a day (S) Daily (D) Once every few days (F) Once a week (W) Never (N) 3. I found the e-learning platform easy to use. (1 = strongly disagree; 6 = strongly agree) 4. I experienced few or no technical problems when trying to access the e-learning platform (Moodle). (1 = strongly disagree; 6 = strongly agree) An e-learning platform (Moodle) can be seen to have four main functional areas: • *Information (announcements, course information, calendar, etc.)*
• *Content (weekly study material, answers, links, etc.)*
• *Communication (discussion forums with lecturers/facilitators or fellow students, etc.)*
• *Assessment (self-tests, assignments, examination guidance, etc.)*
5. Please rank the e-learning platform (Moodle) features you used during the course from least helpful to most helpful. (Rank from 1 – least helpful to 4 – most helpful) E-learning platform Information Content Communication Assessment 6. The discussion forums on the e-learning platform were adequate to be able to share information and extend my understanding of the materials in the course. (1 = strongly disagree; 6 = strongly agree) 7. Did you experience problems with the discussion forums? Yes (Y)/no (N) 8. In future, would you like to see further development of and delivery through online self-study? Yes, I would like to spend more time for online self-study and have less face-to-face contact through lectures (Y). No, I would prefer to go back to more face-to-face teaching (N). I am happy with the current mixture of online and face-to-face contact time (H).
